# Hydroxyapatite-Based Solution as Adjunct Treatment for Biofilm Management: An In Situ Study

**DOI:** 10.3390/nano11092452

**Published:** 2021-09-21

**Authors:** Cíntia M. G. Nobre, Belinda König, Norbert Pütz, Matthias Hannig

**Affiliations:** Clinic of Operative Dentistry, Periodontology and Preventive Dentistry, Saarland University Hospital, 66421 Homburg, Germany; Belinda.Koenig@uks.eu (B.K.); Norbert.puetz@uks.eu (N.P.); Matthias.Hannig@uks.eu (M.H.)

**Keywords:** nanomaterials, biomaterials, oral biofilm, hydroxyapatite

## Abstract

Synthetic hydroxyapatite-based solution is a bioinspired material that may present anti-adhesive properties, restraining the dental biofilm formation without causing adverse effects. This in situ study aims to evaluate the effects of three different hydroxyapatite (HAP) watery solutions as a mouthwash against biofilm adhesion on different dental material surfaces under oral conditions. Hence, four volunteers carried maxillary splints containing enamel, titanium, ceramics, and polymethyl-methacrylate resin (PMMA) samples. Three HAP watery solutions (5%) were prepared with HAP particles presenting different shapes and sizes (HAP I, HAP II, HAP III). During 24 h, the volunteers rinsed two times with one of the following selected tested solution: HAP I, HAP II, HAP III, water, or chlorhexidine 0.2% (CHX). The first rinse was performed 3 min after pellicle formation; the second rinse occurred after a 12 h interval. The surface analysis was performed by scanning electron microscopy (SEM), fluorescence microscopy (FM), and transmission electron microscopy (TEM). Statistical and microscopic analysis showed that most samples treated with any HAP solution revealed reduced biofilm coverage presenting comparable results to CHX treated samples, however without altering the microorganisms’ viability. In conclusion, the results of this investigation showed that a pure hydroxyapatite-based mouthrinse could be a promising bioinspired adjunct solution for biofilm management.

## 1. Introduction

The biofilm is a microbial community embedded in an extracellular polymeric matrix. It is composed of various bacterial species adherent to each other [1]. Biofilm formation starts by adherence of bacteria to the acquired pellicle. The acquired pellicle is a proteinaceous film which is formed not only on the natural enamel but also on any surface in a few seconds after exposure to the intraoral environment, such as dental materials used in oral rehabilitation, like restorations, implants, crowns, and prosthetic/orthodontics appliances [1,2]. Therefore, all surfaces in contact to the intraoral environment are prone to biofilm formation.

The oral biofilm is the main etiological agent for common oral diseases such as caries, dental plaque induced gingivitis, periodontitis, and peri-implantitis [1,3,4,5]. Mechanical treatment is the standard strategy to control biofilm formation and accumulation. However, sometimes the mechanical cleaning alone is not enough to provide oral health [6,7]. Thus, to improve the biofilm control, the use of adjunct treatments should be implemented when indicated. 

Fluorides and chlorhexidine (CHX) are well documented adjunct solutions used to prevent caries and to treat periodontal diseases, respectively [8,9]. However, when chronically ingested, fluorides contribute to an increased incidence of dental fluorosis in the developing dentition. Moreover, the long-term use of chlorhexidine is associated with various side effects [8,9]. New strategies have been developed in the field of preventive dentistry to improve biofilm management [10,11]. For a long period, the research in this area was mainly focused on materials with antibacterial properties such as silver, zinc, chitosan, antimicrobial peptides, and others [12,13,14]. The long-term use of these treatments could lead to the emergence of more resistant bacterial strains and or induce a dysbiosis within the oral cavity [2,15].

Therefore, with the advent of new technologies and new knowledge about biofilm formation, several novel approaches are being discussed in the literature. Targeting the formation cascade of biofilms (bacterial attachment, biofilm maturation and biofilm dispersion), new strategies are being studied [15]. Materials presenting anti-adhesive properties are an interesting alternative, since they have no impact on the oral microflora, while avoiding bacterial resistance. Hydroxyapatite (Ca_10_(PO_4_)_6_(OH)_2_) is a calcium-phosphate ceramic and represents more than 90% of the mineral composition of dental enamel [16]. Synthetic HAP is biocompatible, and it has morphological and structural similarities to enamel apatite crystals, with special properties such as high surface energy, high solubility, and optimal bioactivity. Moreover, it is non-toxic and non-immunogenic, presenting no side effects to the human health, thus being an interesting potential bioinspired material [17]. 

Previous in situ studies from Kensche et al. and Nobre et al. observed the anti-adhesive effect of a bioinspired hydroxyapatite (HAP) solution, which significantly decreased the accumulation of bacteria on enamel and on polished titanium surfaces, respectively [18,19]. Another in situ study by Nobre et al. showed that the HAP particles adhered to the pellicle surface on natural enamel and on some dental material surfaces [20]. This arises the question about the possible anti-adhesive effect of HAP particles against biofilm formation also on different dental materials. 

Additionally, recent studies show that the size and shape of the HAP particles can influence its properties and applications, suggesting that the smaller the particle, the better the properties of the hydroxyapatite particles [21,22,23]. Therefore, this in situ study aims to evaluate the effects of three different HAP watery solutions, presenting different sizes and configurations, as a mouthwash against the biofilm adhesion on enamel and on three different dental materials commonly used in oral rehabilitation: titanium, ceramics, and polymethyl methacrylate resin (PMMA). 

## 2. Materials and Methods

### 2.1. Subjects

Four healthy volunteers (28–35 years old) used an intraoral splint to evaluate the biofilm formation on enamel, titanium, ceramic and PMMA. To be part of the experiments, the volunteers had to present no systematic diseases; no smoking habits, absence of orthodontic appliances; oral healthy; and have not used antibiotics or have not made periodontal treatment within the past 6 months. An oral examination was carried out by a dentist and an informed written consent was obtained from all subjects. The study was conducted in accordance with the Declaration of Helsinki, and the protocol was approved by Medical Ethics Committee of the Medical Association of Saarland, Germany (nº 283/03–2016).

### 2.2. Tested Samples

In addition to dental enamel, the natural component of the tooth, the following materials used for oral rehabilitation were addressed: titanium, ceramic and polymethyl methacrylate resin (PMMA).

#### 2.2.1. Enamel Samples

Square enamel slabs (5 mm long, 1 mm height) were prepared from bovine incisors teeth. All the surfaces were polished with sandpapers (240–4000 grit for SEM and TEM analyses). To remove the resulting smear layer, the slabs were treated with ultrasonication in 3% NaOCl for 3 min, followed by ultrasonication with distilled water for 5 min. Afterwards, a disinfection in 70% ethanol for 15 min took place. Finally, samples were washed with sterile water and stored at 4 °C in sterile water during 24 h before exposure to the oral cavity.

#### 2.2.2. Titanium Samples

The titanium (Ti) discs presented a micro structured surface, with Ra = 2 µm, grade 2, diameter 5 mm and height 1 mm (Dentsply Sirona, Bensheim, Germany). They were polished by grinding with abrasive paper (800–4000 grit). Later, to clean and disinfect the Ti samples were placed in the ultrasonicator and a 15 min immersion in isopropanol (70%) took place, followed by ultrasonication with distilled water. At last, the titanium discs were dried before use.

#### 2.2.3. Ceramic Samples

Square/rectangular ceramic slabs (5 mm long, height 1 mm) were cut from feldspathic ceramic blocks (VITABLOCS Mark II from VITA Zahnfabrik, Germany). These slabs were polished with sandpapers (240–4000 grit). For cleaning and disinfection purpose, the samples were placed in the ultrasonicator and immersed in isopropanol (70%) for 15 min, followed by ultrasonication with distilled water and finally dried before use.

#### 2.2.4. PMMA Samples

Polymethyl methacrylate (PMMA) resin samples (diameter 5 mm, height 1 mm) were prepared with an autopolymerizing prosthetic resin kit (powder and monomer) from Paladent^®^ (Kulzer, Germany) in accordance with the manufacturer’s instructions. They were polished with grid sandpapers (1200–4000 grit). For cleaning purpose, samples were first placed three times (10 min each) in the ultrasonicator, two times with isopropanol 70%, and one with sterile water. Finally, the samples were dried before attachment to the splints.

### 2.3. Tested Solutions

Three powders containing hydroxyapatite nanoparticles (HAP) were used to prepare the test solutions (Table 1). In our previously published paper, the particles size and shape were verified by scanning electron microscope (SEM) and transmission electron microscope (TEM) [19]. The HAP containing test solutions were prepared mixing 0.5 g powder in 10 mL bidistilled water. Chlorhexidine 0.2% (0.2% (*w/v*) chlorhexidine-digluconate in 7% (*v/v*) ethanol—Saarland University Hospital Pharmacy, Germany) and water rinsing were used as control solutions. To prevent any interference between the used solutions, the following rinsing protocol was stablished: the first solution used by each volunteer was the water control. One week later, the HAP test solutions were introduced respecting the following order: HAP I, HAP II, and HAP III; and respecting a two-week clearance period between each of them. At least, Chlorhexidine 0.2% (CHX) was used after another 2 weeks interval.

### 2.4. Oral Exposure

**Figure 1:**
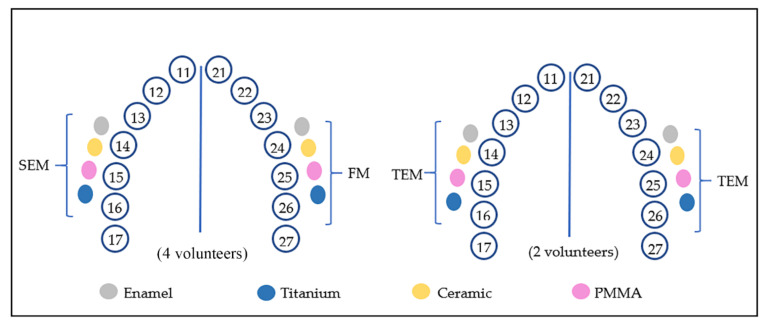
Graphic representation of the samples’ location in the maxillary splint and the microscopic analysis: FM, SEM, and TEM. The TEM analysis was performed in a posterior moment, in which the samples were attached to new splints in two volunteers. This arrangement was reproduced for all tested solutions (water, HAP I, HAP II, HAP III, and CHX).

The volunteers used a customized maxillary splint produced with methacrylate foils (1.5 mm thick) and extending from premolars to the first molar. The buccal region of the splints was perforated to provide the fixation of the polyvinyl siloxane impression material with the attached samples. In the first step of this experiment, one sample of each material was mounted in each upper quadrant, totalizing 8 samples per volunteer for each rinsing solution: 4 samples to be analyzed by FM and another 4 samples by SEM. Therefore, considering the total of 4 volunteers, 16 samples were analyzed with SEM and 16 samples with FM for each one of the five tested solutions, and 4 samples for each tested substrate per test solution (Figure 1).

Each volunteer brushed their teeth with toothbrush and tap-water only, to avoid interferences from the compounds of toothpastes. Right after, the intraoral splits were placed. After three minutes of intraoral exposure, the volunteers performed the first 30 s rinsing with 10 mL of the selected solution (water, HAP I, HAP II, HAP III or CHX). The second rinse was performed 12 h after. The samples were kept in the oral cavity for 24 h.

During the experiment, the volunteers took off the intraoral appliance during meals. They were advised to brush their teeth without toothpaste or any kind of mouthwash after each meal and place the splints again after 10 min. During meals, the splints were stored in a plastic box at 100% humidity and room temperature.

After 24 h, the samples were removed from the splints and immediately rinsed with distilled running water to remove the non-adsorbed salivary film. The samples were prepared for fluorescence microscope (FM) and scanning electron microscope (SEM). The same protocol was repeated by 2 volunteers, who participated in the first experiment, to prepare eight additional samples (Figure 1) for transmission electron microscopy (TEM).

### 2.5. Fluorescence Microscopy: Biofilm Coverage and Viability Assay

Briefly, the fluorescence microscopy analysis allows to detect, quantify, and differentiate the living from the dead microorganisms in the biofilm. The staining kit used (LIVE/DEAD^®^ BacLight™ Bacterial Viability kit L7012, Invitrogen—Thermo Fisher Scientific, Carlsbad, CA, USA) consists in mixing the following two nucleic acid stains: the SYTO 9, which stains all bacteria in green, and propidium iodide, which stains only cells with compromised membranes in red [24]. The staining was used for the determination of cell viability and to evaluate the biofilm coverage, allowing a relative comparison for membrane damage within the test groups. For full details about the staining procedure, see Nobre et al. [19]. 

To perform both evaluations, 9 pictures per sample at 1000-fold magnification were taken under the fluorescence microscope. The Image J (ImageJ2, National Institutes of Health, LOCI, University of Wisconsin, USA), was used to do the viability correlation. The software allows to measure the integrated density of the red (dead/damaged cells) and green (living cells) channel from each FM micrograph. Then, the total density and the percentage of live cells could be calculated (viability assay).

The semi quantitative analysis of the biofilm coverage was made with Sefexa Image Segmentation Tool (www.fexovi.com/sefexa.html, accessed 15 January 2019). First, the FM micrograph containing both channels is converted into grayscale and then, the program measures the total area of the picture. The biofilm area can be selected, separated from the background, and calculated. Finally, with a proportion calculation, it is possible to determine the biofilm coverage percentage. A medium coverage per sample was calculated using all FM pictures from one sample.

### 2.6. Scanning Electron Microscopy

The detection of adhered HAP particles and the investigation of the bacterial coverage was performed by SEM analysis. All samples were prepared according to ours previously published protocol, which can be found in Nobre et al. [19]. Except the drying process, in which the samples were left in the air chamber overnight. After drying, samples were attached to aluminum stubs, and sputter-coated with carbon. SEM evaluations were made in a XL30 ESEM FEG (FEI, Eindhoven, The Netherlands) at 5 kV at up to 20,000-fold magnification.

### 2.7. Transmission Electron Microscopy

TEM analysis was performed on specimens carried by two volunteers in order to visualize the ultrastructure of the biofilm. After removal of the splints, all the four different samples followed a rigorous protocol according to the methods in Nobre et al. [19]. Except that in the present experiment, the following additional procedure was performed only for titanium and ceramics samples: after polymerization, titanium and ceramics were removed by treatment with hydrofluoric acid (5%) during 48 h, and the specimens were re-embedded in Araldite. The enamel was decalcified due to exposure in 0.1M HCl for 4 h, and the specimens were re-embedded in Araldite.

As the final step, the specimens were cut in ultra-thin sections in an ultramicrotome with a diamond knife (Leica EM UC7, Germany) and mounted on Pioloform-coated copper grids and contrasted with aqueous solutions of uranyl acetate and lead citrate at room temperature. After intensive washing with distilled water, biofilms were analyzed with a TEM Tecnai 12 Biotwin (FEI, Eindhoven, The Netherlands) under a magnification up to 100,000-fold.

### 2.8. Statistic

In this study, the qualitative (SEM and TEM figures) and quantitative (FM figures) methods were applied. Quantitative assessment of the FM micrographs was performed with the GraphPad Prism 6 software, which was used to analyze the mean values. Two-way RM ANOVA with Tukey’s correction for multiple comparison test was used during the statistical evaluation to:

- Evaluate the difference of the same material in the different rinsing solutions for coverage and viability tests
- Assess the difference between each material when the same solution was used to compare the HAP anti-adhesive properties against biofilm for each dental material

The Statistical significance was considered for *p* < 0.05. 

## 3. Results

### 3.1. Fluorescence Microscopic Analysis: Biofilm Coverage and Viability Assay

Biofilms were formed within 24 h of oral exposure on all samples, regardless of the solution used. Fluorescence microscope investigation allowed the visualization of microorganism coverage and microorganism cells viability, where the living cells were represented by green fluorescence and dead cells, by red. 

**Figure 2:**
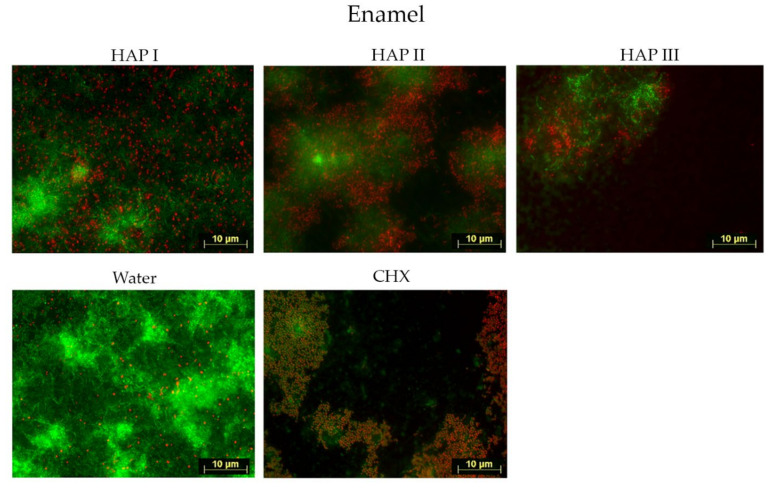
Viability assay: fluorescence microscopic investigation of the stained 24 h biofilm on enamel slabs after two times rinsing with HAP I, HAP II, HAP III, CHX and water. Water control samples are densely covered with live microorganisms’ cells and a few single dead cells. The specimens rinsed with CHX presented mostly dead microorganisms, but also some green colonies were visible. HAP rinsed samples presented a similar pattern, with mainly living microorganisms but surrounded by single dead cells or small colonies of dead cells.

**Figure 3:**
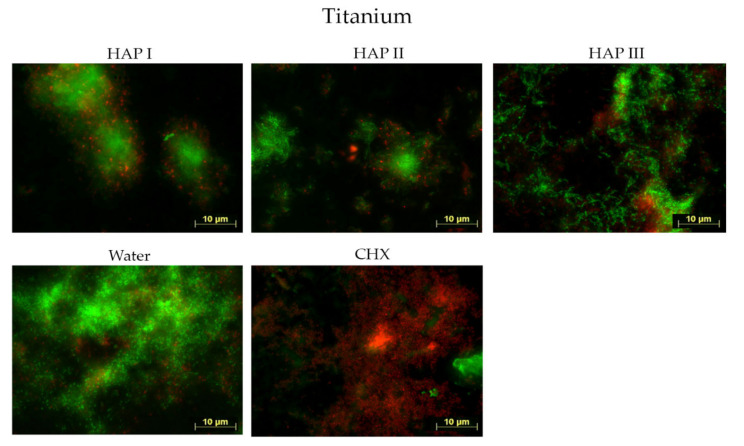
Viability assay: fluorescence microscopic investigation of the stained 24 h biofilm on titanium after two times rinsing with HAP I, HAP II, HAP III, CHX and water. The samples rinsed with HAP solutions presented mostly live microorganisms agglomerated in colonies. When water solution was used, an increased quantity of microorganisms is visible covering a bigger area of the micrograph. The specimens rinsed with CHX presented mostly dead cells.

**Figure 4:**
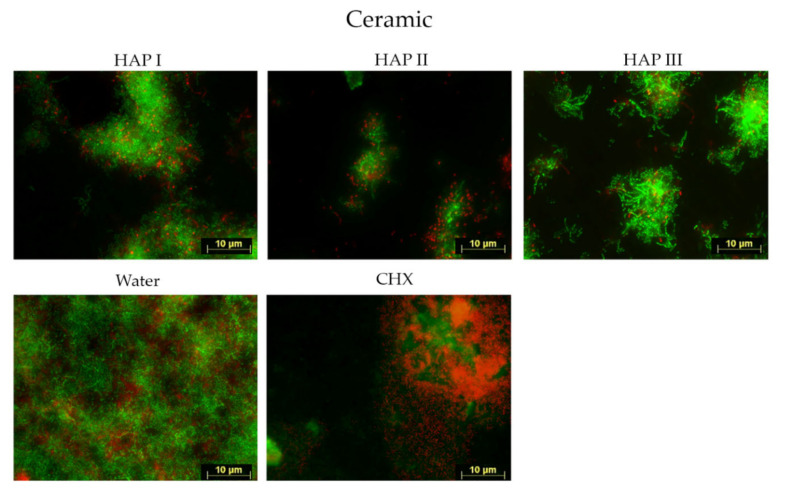
Viability assay: fluorescence microscopic investigation of the stained 24 h biofilm on ceramic after two times rinsing with HAP I, HAP II, HAP III, CHX and water. A lower number of microorganisms were found when HAP solutions were applied, characterized by the presence of green islands of cells surrounded by red single cells.

**Figure 5:**
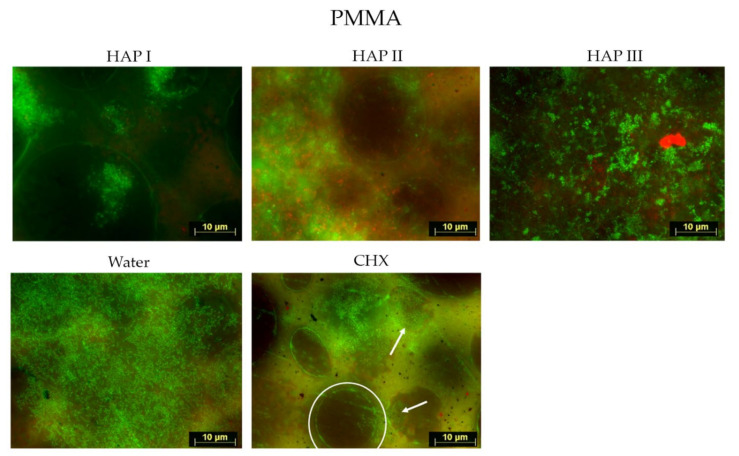
Viability assay: fluorescence microscopic investigation of the stained 24 h biofilm on PMMA after two times rinsing with HAP I, HAP II, HAP III, CHX and water. These samples presented slightly more microorganisms than all the other materials. Colonies of living cells are also visible when chlorhexidine was applied (white arrows). The superficial layer of the PMMA surface was usually stained in red or in green, which is visible on micrographs from HAP I, HAP II and CHX. The white circle delimitates the prepolymerized PMMA particles.

**Figure 6:**
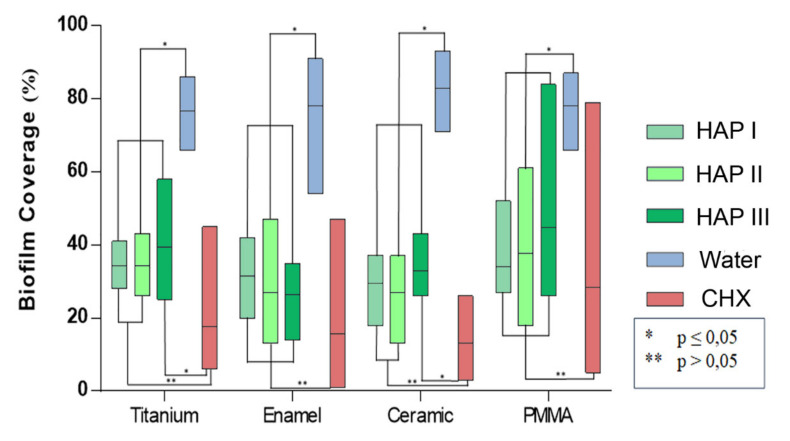
Twenty-four hour biofilm coverage on different dental material evaluated under FM. Samples rinsed with water presented a significant denser biofilm than samples rinsed either with CHX or HAP solutions (*p* < 0.0001). A significantly lower number of microorganisms was detected when CHX rinse was applied compared with water rinsed samples (*p* = 0.0099). However, most samples treated with the HAP solutions showed a lower biofilm coverage, without a significant difference from the CHX rinsed samples, except for titanium and ceramic samples rinsed with HAP III. Enamel, titanium, and ceramics specimens presented a lower quantity of microorganisms than PMMA for all HAP solutions, without significant difference between them. This figure belongs to the thesis of C.M.G. Nobre.

Concerning the biofilm coverage, samples rinsed with water presented a significantly higher percentage of coverage than samples rinsed with any other solutions tested (*p* < 0.0001) (Figure 2, Figure 3, Figure 4, Figure 5 and Figure 6). When CHX rinse was applied, a significant lower number of microorganisms could be detected compared with the water control (*p* = 0.0099). On the other hand, most samples treated with any of the three HAP solutions showed lower biofilm coverage, without significant difference from CHX treated samples, except for the titanium and ceramic samples rinsed with HAP III. Difference concerning the materials applied in this study was subtle. Ceramics and titanium specimens presented an inferior quantity of microorganisms than enamel or PMMA when rinsing with any HAP solution. 

**Figure 7:**
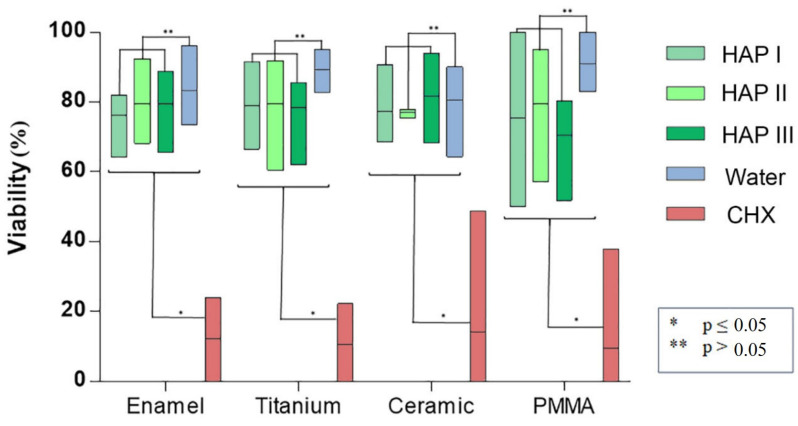
Bacteria viability results of the 24 h biofilm formed on different dental materials evaluated under FM. There was a significant difference when comparing the samples rinsed with any of the HAP solutions with the samples rinsed with CHX (*p* < 0.0001), where most cells were dead. There was no significant difference between the samples rinsed with the HAP solutions and the samples rinsed with water. Additionally, there was no significant difference in microorganism’s viability between the applied materials when the same rinsing solution was used. This figure belongs to the thesis of C.M.G. Nobre.

Concerning the biofilm viability, samples rinsed with any of the HAP solutions had a higher number of live microorganisms, which had significant difference with CHX rinsing samples (*p* < 0.0001), where most microorganism were dead. No significant difference was found between rinsing with HAP solutions and samples rinsed with water. After rinsing with both types of solutions, samples presented most living cells, stained in green (Figure 2, Figure 3, Figure 4 and Figure 5 and Figure 7). Additionally, independent of the solution used as mouthrinse, there was no significant difference on the viability between the tested materials, when the same rinsing solution was applied.

### 3.2. Scanning Electron Microscopic Analysis

**Figure 8:**
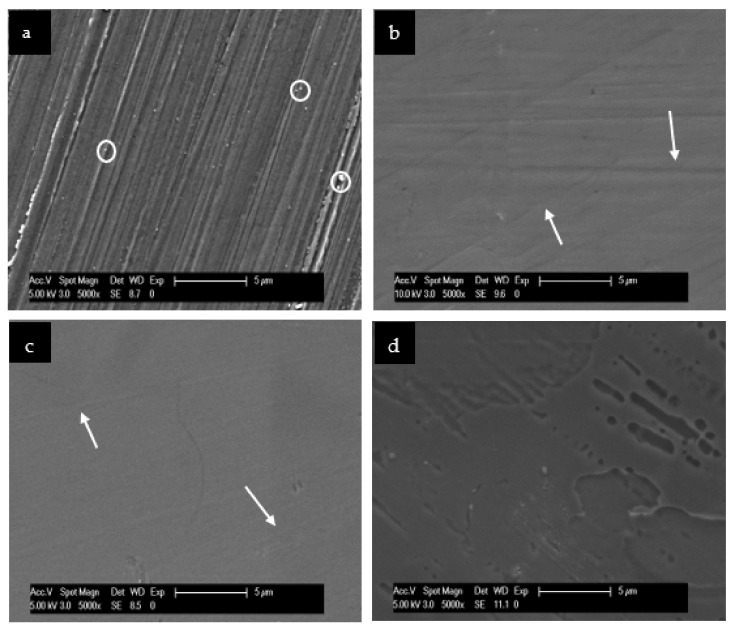
SEM analysis at 5000-fold magnification of the tested surfaces after the polishing procedure: enamel (**a**), titanium (**b**), ceramic (**c**) and PMMA (**d**). (**a**) The white dots (white circle) visible over the enamel surface may represent debris from the polishing process. (**b**) and (**c**): The white arrows show some scratches on titanium and ceramic surfaces, which resulted also from the polishing process. (**d**) The micrograph from PMMA surface shows a “crater-like” pattern, which is commonly visible on these surfaces, probably representing the prepolymerized PMMA particles.

**Figure 9:**
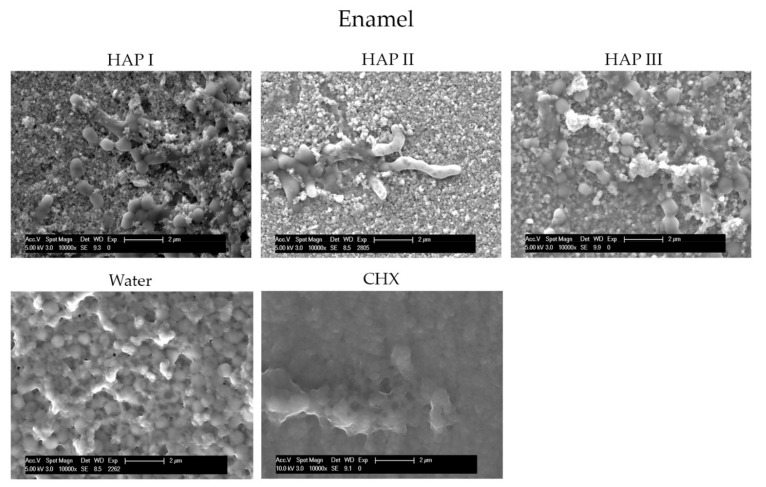
SEM analysis at 10,000-fold magnification shows the differences of biofilm amount on the enamel surface after 24 h of intraoral exposure and two times rinsing with HAP I, HAP II, HAP III, CHX and water, respectively. The white arrows are pointing to the bacteria; the white asterisk represents the HAP particles. On samples rinsed with HAP solutions the material surface is covered with a thick globularly pellicle layer, bacterial cells, and HAP particles.

**Figure 10:**
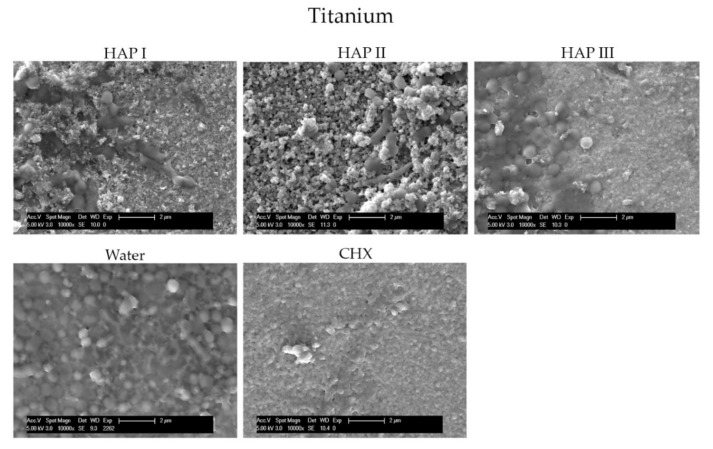
SEM analysis at 10,000-fold magnification shows the differences on biofilm amount on the titanium surface after 24 h of intraoral exposure and two times rinsing with HAP I, HAP II, HAP III, CHX and water. The white arrows are pointing to the coccoid shaped bacteria; the white asterisk represents the HAP particles. On samples rinsed with HAP solutions the material surface is covered with a thick globularly pellicle layer, bacterial cells, and HAP particles. The pellicle layer can also be observed on samples rinsed with CHX.

**Figure 11:**
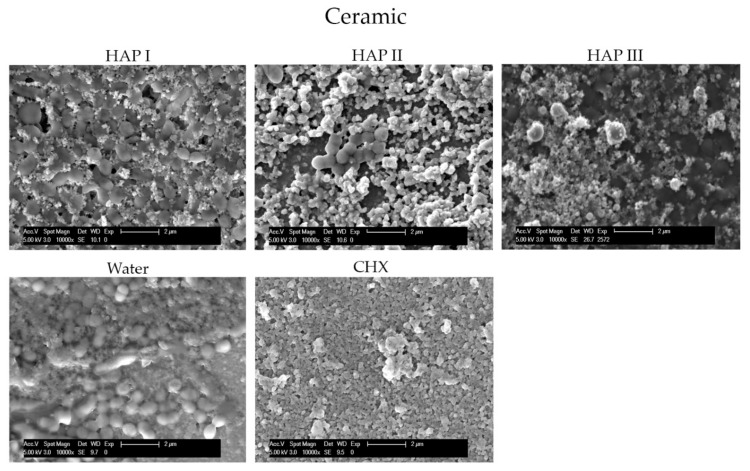
SEM analysis at 10,000-fold magnification shows the differences on biofilm amount on the ceramic surface after 24 h of intraoral exposure and two times rinsing with HAP I, HAP II, HAP III, CHX and water. The white arrows are pointing to the coccoid shaped bacteria; the white asterisk represents the HAP particles. On samples rinsed with HAP solutions the material surface is covered with a thick globularly pellicle layer, bacterial cells, and HAP particles.

**Figure 12:**
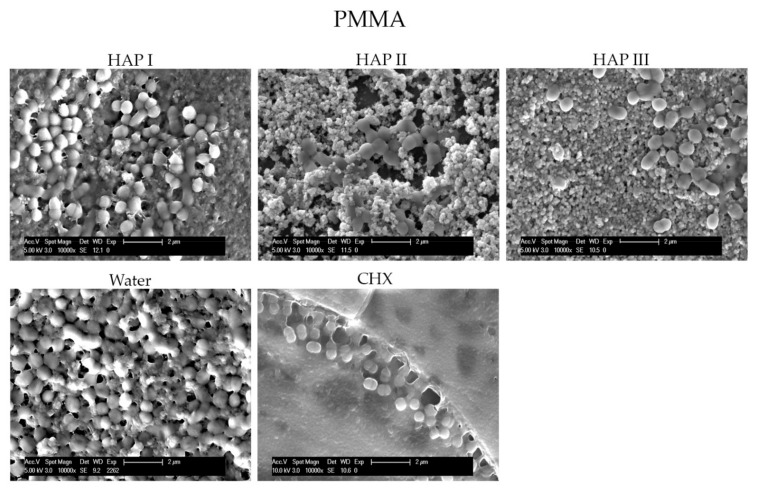
SEM analysis at 10,000-fold magnification shows the differences on biofilm amount on the PMMA surface after 24 h of intraoral exposure and two times rinsing with HAP I, HAP II, HAP III, CHX and water. Bacterial colonies were also visible on samples rinsed with CHX. They were located in retention grooves of the PMMA surface. The white arrows are pointing to the coccoid shaped bacteria; the white asterisk represents the HAP particles.

To understand and evaluate the biofilm coverage on each surface properly, it is important to visualize their characteristics immediately after the polishing process, without intraoral exposure, which can be seen on Figure 8. After 24 h of intraoral exposure, the formed biofilm consisted mainly of coccoid bacteria, independent of the dental material or rinsing solution used. Within the biofilm, it was possible to observe under SEM that these bacterial cells were dispersed on the different samples’ surfaces as individual cells or as colonies (Figure 9, Figure 10, Figure 11 and Figure 12). 

The micrographs presented in Figure 9, Figure 10, Figure 11 and Figure 12 showed that the SEM investigations corroborated the fluorescence microscopy results. A dense, and multilayered biofilm was visible on samples rinsed with water, whereas only few isolated bacterial cells or small conglomerates were visible when rinsing was performed with CHX. 

Specimens rinsed with any of the HAP solutions revealed considerably less biofilm than the water control samples. It was also possible to visualize a dense globular layer, representing the formed oral pellicle. The hydroxyapatite particles formed bigger and small agglomerates on top of the bacteria cell and dispersed on the pellicle layer. The smaller HAP clusters also presented a globular shape; thus, it was not possible to distinguish the pellicle’s globular particles from the small agglomerates of hydroxyapatite. Furthermore, the PMMA samples presented more biofilm on their surface in comparison to the other materials (Figure 12). 

**Figure 13:**
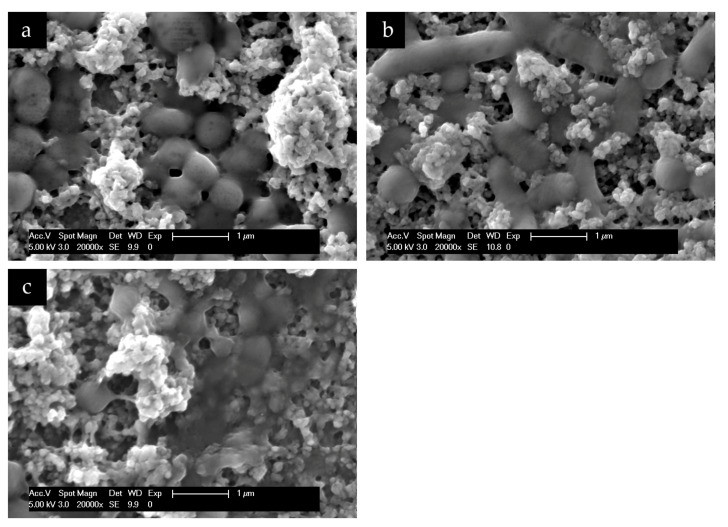
SEM analysis at 20,000-fold magnification of HAP I (**a**), HAP II (**b**) HAP III (**c**) particles attached on the 24 h biofilm formed on titanium (**a**), PMMA (**b**), and enamel (**c**), under the effect of two times rinsing with the respective HAP solution. Agglomeration and single particles of HAP particles are visible on the bacteria surface. The HAP particles seems attached to the bacterial cell, showing a possible bacteria-hydroxyapatite interaction through the presence of connective structures (white arrows).

Additionally, SEM evaluation at higher magnifications enabled the visualization of a bridge-like structure, indicating a possible interaction between the hydroxyapatite particles and bacterial cells (Figure 13).

### 3.3. Transmission Electron Microscopic Analysis

Considering the PMMA samples, it was not possible to visualize any biofilm structure by TEM with the methodology applied. Therefore, TEM evaluation was performed only with enamel, titanium, and ceramics samples. 

**Figure 14:**
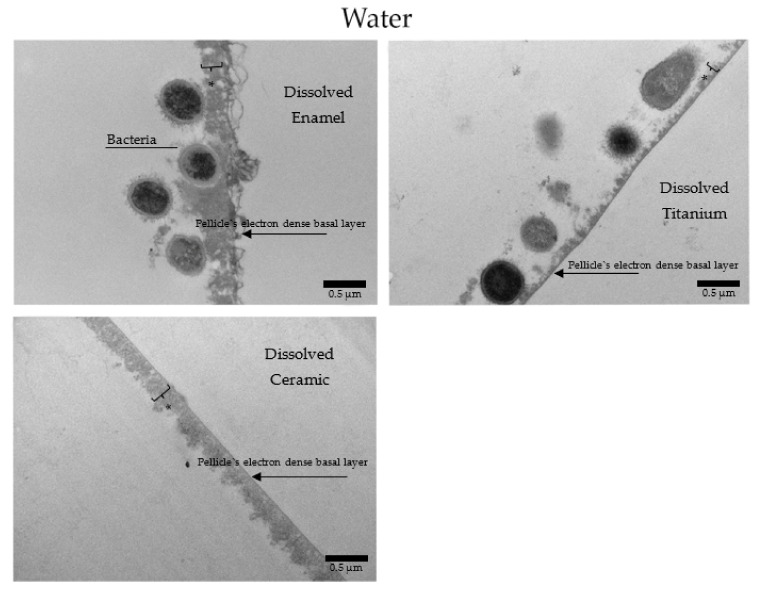
TEM micrographs at 30,000-fold magnifications of a 24 h biofilm on enamel, titanium, and ceramic surfaces after water rinsing. Bacterial cells are adhered onto the pellicle formed on all surfaces. Fimbriae could be observed on the bacteria surface. The asterisks represent the pellicle’s outer layer.

**Figure 15:**
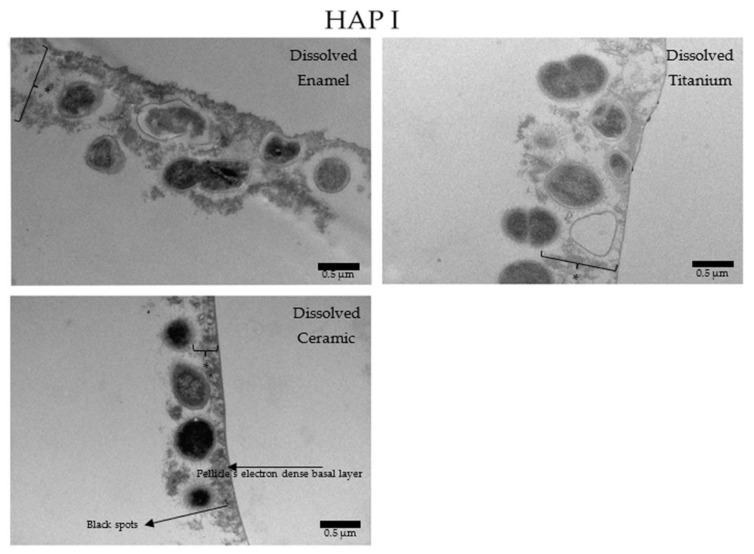
TEM micrographs at 30,000-fold magnifications of a 24 h biofilm formed on enamel, titanium, and ceramic surfaces after rinsing with HAP I. The asterisks represent the pellicle’s outer layer. Some small black spots scattered randomly in the sample were detected. They may represent single particles of hydroxyapatite nanoparticles that were not dissolved during the TEM processing steps.

**Figure 16:**
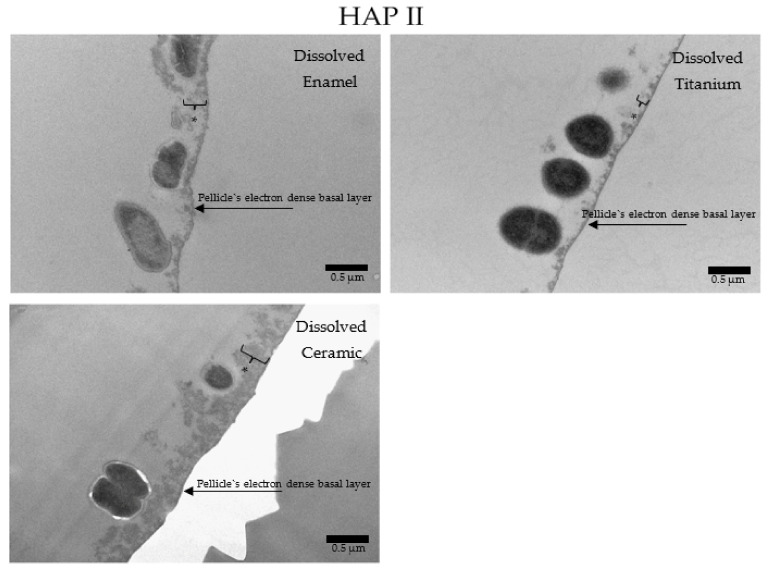
TEM micrographs at 23,000-fold (Ti) and 30,000-fold (enamel and ceramic) magnifications of a 24 h biofilm formed on enamel, titanium, and ceramic surfaces after rinsing with HAP II. Attached bacteria are visible on all surfaces. The asterisks represent the pellicle’s outer layer.

**Figure 17:**
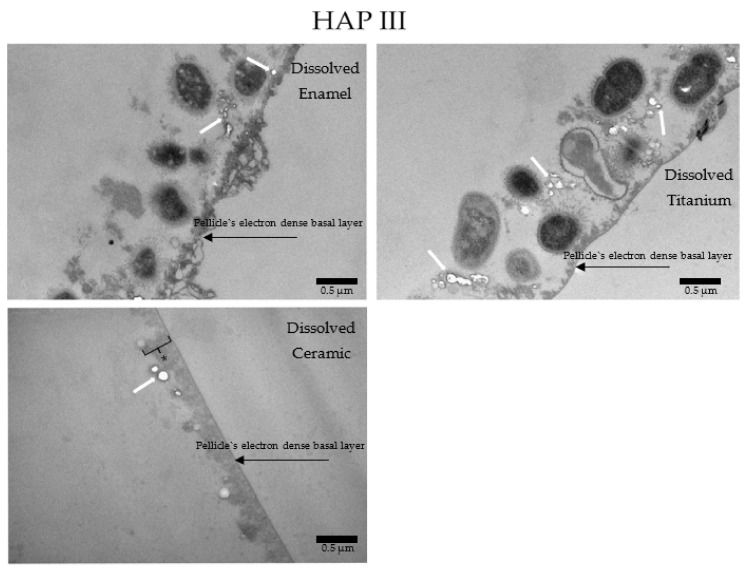
TEM micrographs at 30,000-fold (enamel and Ti) and 49,000-fold (ceramic) magnifications of a 24 h biofilm formed on enamel, titanium, and ceramic surfaces after rinsing with HAP III. It is possible to visualize round-shaped structures of low electron density (white arrows). They might represent HAP particles that were dissolved during the TEM processing steps (ultrathin sectioning). The asterisks represent the pellicle’s outer layer.

Under the TEM micrographs at 30,000-fold magnification it is possible to visualize that samples rinsed with water present a higher number of bacterial cells (Figure 14), presenting the typical 24 h pellicle ultrastructure with an electron dense basal layer and a granular, globular outer layer. Depending on the materials applied, some differences on the characteristics of the pellicle’s ultrastructure were visible. Enamel samples presented a heterogeneous, diffuse, and not well-defined basal layer, while this same layer on ceramics and titanium samples was thicker and appeared as a clear line in contact with the material surface. On enamel, the granular second outer layer was a thick and loose structure. On ceramics, the outer layer was also thick, but very compact. On titanium, this second layer was thin and very dispersed. Another similar finding of all samples rinsed with water was the presence of a mono or double layer of integrated bacteria colonizers on top of the pellicle’s outer layer, representing the 24 h biofilm. Is possible to visualize filiform structures around the bacteria, representing their mechanism of adhesion (Figure 14, Figure 15, Figure 16 and Figure 17). 

When HAP I (Figure 15), HAP II (Figure 16) or HAP III (Figure 17) were used, the samples followed a similar pattern concerning the pellicle’s basal and outer layer for each material. Additionally, there were peculiarities concerning each hydroxyapatite solution. Samples rinsed with HAP I or HAP II presented some black spots randomly scattered on the pellicle surface and within the pellicle, which could represent residues from single particles and clusters of hydroxyapatite nanoparticles. Differently, on all samples rinsed with HAP III brighter and round-shaped structures were detected. These structures might be the hydroxyapatite particles that were dissolved during the ultrathin sectioning process. Furthermore, most of the bacteria presented changes on their inner morphology, containing these same brighter and round-shaped structures.

**Figure 18:**
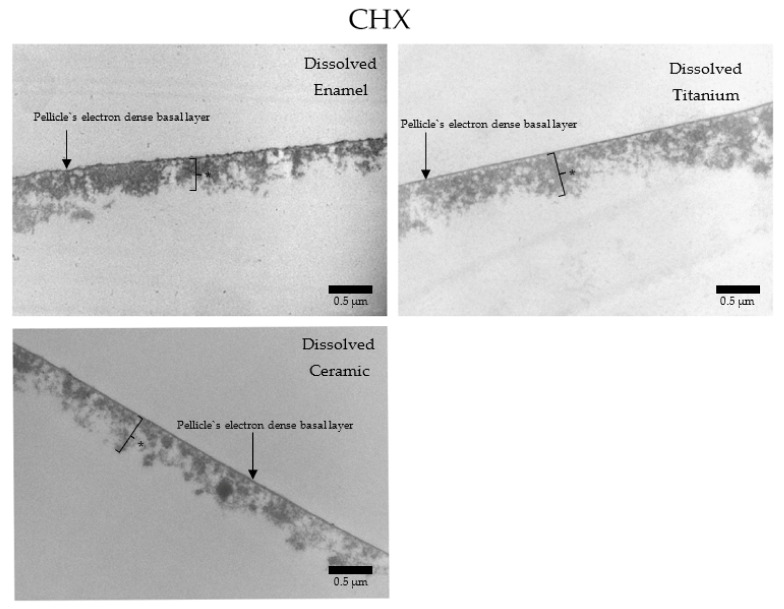
TEM micrographs at 30,000-fold magnifications of a 24 h pellicle formed on enamel, titanium, and ceramic surfaces after rinsing with CHX. Pellicle on all three materials present a thick basal and outer layer, with no visible adherent bacteria. The asterisks represent the pellicle’s outer layer.

In contrast, when CHX was applied (Figure 18), a similar pellicle ultrastructure was present on enamel, titanium, and ceramics. The basal and the second layers were thicker (c. 300–2000 nm) and more electron dense without the presence of adherent bacteria.

## 4. Discussion

For the first time, this in situ investigation evaluated the effects of three different hydroxyapatite particles applied as oral rinsing solutions on the biofilms formed on enamel, titanium, ceramic, and PMMA. Our study revealed a promising anti-adherent effect for the HAP mouthrinse, regardless of the shape and size of the HAP nanoparticles and the dental materials included in this study.

In accordance with our previously published study, the in situ model was applied due to its capacity to represent the natural oral environment, thus better reproducing the oral pellicle and the biofilm formation cycle, comprehending a dynamic and multifactorial process [1,19]. As successfully applied in previous studies, these experiments were performed with intraoral splints with attached samples [18,19,20,25,26,27]. The volunteers carried the intraoral splints for 24 h, and the microscopic analysis was performed with FM, SEM, and TEM. As showed in the results section, it was unviable to analyze the biofilms formed on PMMA samples by TEM. The PMMA surface with the adherent biofilm might have been dissolved in the acetone–araldite mixture during the embedding procedure, failing to make it possible to visualize any structure by TEM microscopy with this methodology applied on PMMA samples. Therefore, TEM evaluation was performed only with enamel, titanium, and ceramics samples.

Few in situ studies showed the anti-adhesive effect of HAP particles on enamel and titanium surfaces [18,19]. In order to understand this interesting property, our previous study revealed a possible interaction between the proteins from the 2 h acquired pellicle formed on different materials and the hydroxyapatite particles through the presence of bridge-like structures between them. This finding demonstrated that nano-HAP can adhere not only to the pellicle formed enamel but also to artificial dental surfaces under oral conditions [20]. The presence of this HAP-pellicle interaction might be the reason behind the incorporation of the hydroxyapatite particles within the pellicle, which resulted in the dense globular structure visible on Figure 9, Figure 10, Figure 11 and Figure 12, making it difficult to distinguish the HAP particles from the pellicle structures. 

The HAP-pellicle interactions observed in our previous study had an impact on the biofilm formation on the same analyzed materials in this study, providing interesting results regarding the HAP solution anti-adhesive effect [20]. After 24 h of intraoral exposure, a multilayered biofilm could be seen covering the surfaces rinsed with water in SEM and TEM figures (Figure 9, Figure 10, Figure 11 and Figure 12 and Figure 14). Interestingly, when HAP I, HAP II or HAP III were used as rinsing solutions, SEM micrographs showed that all the materials surfaces were covered with a similar distribution of adherent hydroxyapatite nanoparticles, presenting a smaller number of microorganisms when compared to samples rinsed with water (Figure 9, Figure 10, Figure 11 and Figure 12). Quantitative analysis with FM confirmed that samples rinsed with HAP I, II and III presented a significantly smaller percentage of coverage than samples rinsed with water (Figure 6). Furthermore, the results also revealed that HAP solutions reduced the biofilm coverage, presenting no significant difference when compared to CHX (Figure 6). This demonstrates that oral rinsing with HAP (5%) solution reduced the number of adherent microorganisms on enamel and on the three different dental material surfaces. Kensche et al. and our group had already shown similar results for enamel and titanium surfaces, consecutively [18,19]. 

SEM micrographs from the samples rinsed with CHX presented a dense pellicle layer and areas without any microorganisms (Figure 9, Figure 10, Figure 11 and Figure 12 and Figure 18), corroborating with the well-known antibacterial properties of the chlorhexidine [9]. Fluorescence microscopy results also demonstrated that application of CHX as a mouthwash presented an efficient bactericidal effect (Figure 2, Figure 3, Figure 4, Figure 5, Figure 6 and Figure 7), significantly reducing the biofilm coverage and viability on all tested surfaces compared to water and hydroxyapatite solutions, which is in accordance with previous publications [18,25,27]. On the contrary, there was no significant difference in viability when comparing the three HAP solutions with water under the fluorescence microscope (Figure 7). No differences in viability between each HAP solution were found either. Thus, both the water solution and the HAP solutions presented most living cells on FM micrographs. These data show that the 5% hydroxyapatite watery solutions had no anti-bacterial effect, regardless of their size or configuration. 

Additionally, findings from TEM analysis of the 24 h biofilm indicated that the three HAP solutions did not affect the pellicle’s ultrastructure, showing similar characteristics as the regular pellicle formed on samples rinsed with water (Figure 15, Figure 16 and Figure 17). Thus, the results from the present study confirmed the data in the literature that HAP solution may have rather an anti-adhesive than anti-bacterial effects [18,19,25,28]. 

The mechanism behind the anti-adherent property of the hydroxyapatite particles solution is not well elucidated in the literature. Kensche et al. stated that the hydroxyapatite nanoparticles accumulated on the enamel surface could prevent the link between bacteria and pellicle receptors by blocking cell wall adhesins from bacteria [18]. On the other hand, some proteins from saliva, such as histatins, have a high affinity to hydroxyapatite crystals present on natural teeth, starting the acquired pellicle formation process [29]. Based on the previously results from our group, showing that HAP nanoparticles could interact with the pellicle surface through bridge-like structures [20], we hypothesized that the same interactions might attract the HAP particles absorbed by the saliva, initiating their attachment onto the pellicle. 

Interestingly, another kind of connective structure was visible in the present study on SEM micrograph under higher magnification, but now between the HAP particles and the bacterial cells (Figure 13). Pepla et al. reported that nano-HAP could bind not only to proteins, but also to the bacteria cells integrated on the biofilm due to their nanosized and consequently increased surface area [30]. Therefore, due to the bridge-like structures visualized in these experiments, we suppose that all the previous hypotheses may occur simultaneously, which resulted in less bacteria on samples rinsed with HAP solutions. The mechanisms and mode of action behind these interactions still needs to be elucidated in detail with further in vivo/in situ studies. 

According to the scarce literature in this matter, the anti-adherent effect might be related to the hydroxyapatite particles sizes. The size effect would facilitate the direct interaction with the bacteria. In other words, smaller particles, such as nano and micro hydroxyapatite particles, would better interact with adhesins on the bacterial membrane, blocking and reducing the attachment of bacterial cells [19,30,31]. Therefore, the initial hypothesis was that HAP I would present better results, however, the three hydroxyapatite solutions presented similar effects in reducing bacterial coverage. This might be related to variations on the particles’ sizes of each hydroxyapatite powder applied in this study, revealing a certain similarity between them. Therefore, the influence of the HAP size-effect on the anti-adhesive properties could not be properly evaluated. 

In general, the anti-adhesive efficacy of hydroxyapatite nanoparticles is promising. However, we must consider the limitations of the present study, such as the considerable small number of subjects, which can be justified by the quantity of materials involved and the complexity of in situ methodologies, as in previous studies with similar methods [18,32,33,34,35]. Another limitation would be that only healthy adults were included. Therefore, the effects of HAP solution were not tested on patients with periodontitis or peri-implantitis. Those patients could represent a challenge due to the presence of deep pockets on probing and calculus formation, where the bacterial biofilm present a more complex environment and it is hard to reach. Additionally, all the materials used went through a polishing process to standardize the surface roughness. This aspect also represents a limitation, since the changes in the surface properties might have influences in their biological effect, reducing the practical application of the used polished surfaces. However, despite the limitations of this study, it was possible to gain insight into the effects of different preparations of HAP solutions on the initial biofilm control. Long-term clinical trials with a larger number of volunteers presenting different stages of periodontal health and biofilm formation should be conducted to comprehend the properties of pure HAP solution.

## 5. Conclusions

The pure hydroxyapatite nanoparticles 5% solutions had a great impact on the oral biofilm under intraoral conditions on enamel, titanium, ceramic and PMMA surfaces. They reduced the biofilm coverage on all tested materials without altering the bacterial viability, which can reduce the risk of a dysbiosis of the oral ecology when compared to other antibactericidal risings such as CHX. Furthermore, the different HAP sizes and morphology used in these experiments had no significant influence on their anti-adherent effects. Due to anti-adherent properties, the HAP efficacy to reduce the initial biofilm coverage was comparable to chlorhexidine 0.2% on all tested surfaces. It is important to mention that the mechanical treatment is still the standard and main approach for the control of dental biofilms. Adjunct solutions can be used as an additional treatment when mechanical elimination of biofilm proves to be difficult. Therefore, the results of this investigation yield the pure hydroxyapatite mouthrinse as a promising bioinspired adjunct solution for biofilm management.

## Figures and Tables

**Table 1:** Specifications of hydroxyapatite particles powders according to manufacturer’s information.

	Company	Country	Median Size	Configuration
HAP I	Eprui	China	40 nm	Needle
HAP II	Kalichem	Italy	100 nm	Needle
HAP III	Sigma Aldrich	Germany	<200 nm	Spherical

## Data Availability

The data presented in this study are available on request from the corresponding author. The data are not publicly available due to privacy.

## References

[1] Hannig C., Hannig M. (2009). The oral cavity—A key system to understand substratum-dependent bioadhesion on solid surfaces in man. Clin. Oral Investig..

[2] Sterzenbach T., Helbig R., Hannig C., Hannig M. (2020). Bioadhesion in the oral cavity and approaches for biofilm management by surface modifications. Clin. Oral Investig..

[3] Busscher H., Rinastiti M., Siswomihardjo W., Van der Mei H. (2010). Biofilm formation on dental restorative and implant materials. J. Dent. Res..

[4] Arweiler N.B., Netuschil L. (2016). The oral microbiota. Microbiota Hum. Body.

[5] Chapple I.L., Mealey B.L., Van Dyke T.E., Bartold P.M., Dommisch H., Eickholz P., Geisinger M.L., Genco R.J., Glogauer M., Goldstein M. (2018). Periodontal health and gingival diseases and conditions on an intact and a reduced periodontium: Consensus report of workgroup 1 of the 2017 World Workshop on the Classification of Periodontal and Peri-Implant Diseases and Conditions. J. Periodontol..

[6] Socransky S.S. (2002). Dental biofilms: Difficult therapeutic targets. Periodontol 2000.

[7] Takenaka S., Ohsumi T., Noiri Y. (2019). Evidence-based strategy for dental biofilms: Current evidence of mouthwashes on dental biofilm and gingivitis. Jpn. Dent. Sci. Rev..

[8] Shaffiey S.R., Shaffiey S.F. (2016). Surface enamel remineralization by biomimetic nano hydroxyapatite crystals and fluoride ions effects. J. Ceram. Process. Res..

[9] James P., Worthington H.V., Parnell C., Harding M., Lamont T., Cheung A., Whelton H., Riley P. (2017). Chlorhexidine mouthrinse as an adjunctive treatment for gingival health. Cochrane Database Syst. Rev..

[10] Hannig M., Hannig C. (2010). Nanomaterials in preventive dentistry. Nat. Nanotechnol..

[11] Elkassas D., Arafa A. (2017). The innovative applications of therapeutic nanostructures in dentistry. Nanomedicine.

[12] Dos Santos V.E., Vasconcelos Filho A., Targino A.G.R., Flores M.A.P., Galembeck A., Caldas A.F., Rosenblatt A. (2014). A new “Silver-Bullet” to treat caries in children–Nano Silver Fluoride: A randomised clinical trial. J. Dent..

[13] Baltzer S.A., Brown M.H. (2011). Antimicrobial peptides–promising alternatives to conventional antibiotics. J. Mol. Microbiol. Biotechnol..

[14] Allaker R.P., Memarzadeh K. (2014). Nanoparticles and the control of oral infections. Int. J. Antimicrob. Agents.

[15] Kuang X., Chen V., Xu X. (2018). Novel approaches to the control of oral microbial biofilms. BioMed Res. Int..

[16] Enax J., Epple M. (2018). Synthetic hydroxyapatite as a biomimetic oral care agent. Oral Health Prev. Dent.

[17] Epple M. (2018). Review of potential health risks associated with nanoscopic calcium phosphate. Acta Biomater..

[18] Kensche A., Holder C., Basche S., Tahan N., Hannig C., Hannig M. (2017). Efficacy of a mouthrinse based on hydroxyapatite to reduce initial bacterial colonisation in situ. Arch. Oral Biol..

[19] Nobre C.M., Pütz N., König B., Rupf S., Hannig M. (2020). Modification of in situ Biofilm Formation on Titanium by a Hydroxyapatite Nanoparticle-Based Solution. Front. Bioeng. Biotechnol..

[20] Nobre C.M.G., Pütz N., Hannig M. (2020). Adhesion of hydroxyapatite nanoparticles to dental materials under oral conditions. Scanning.

[21] Li L., Pan H., Tao J., Xu X., Mao C., Gu X., Tang R. (2008). Repair of enamel by using hydroxyapatite nanoparticles as the building blocks. J. Mater. Chem..

[22] Eliaz N., Metoki N. (2017). Calcium phosphate bioceramics: A review of their history, structure, properties, coating technologies and biomedical applications. Materials.

[23] Huang S., Gao S., Cheng L., Yu H. (2011). Remineralization potential of nano-hydroxyapatite on initial enamel lesions: An in vitro study. Caries Res..

[24] Stiefel P., Schmidt-Emrich S., Maniura-Weber K., Ren Q. (2015). Critical aspects of using bacterial cell viability assays with the fluorophores SYTO9 and propidium iodide. BMC Microbiol..

[25] Hannig C., Basche S., Burghardt T., Al-Ahmad A., Hannig M. (2013). Influence of a mouthwash containing hydroxyapatite microclusters on bacterial adherence in situ. Clin. Oral Investig..

[26] Grychtol S., Basche S., Hannig M., Hannig C. (2014). Effect of CPP/ACP on initial bioadhesion to enamel and dentin in situ. Sci. World J..

[27] Hannig C., Gaeding A., Basche S., Richter G., Helbig R., Hannig M. (2013). Effect of conventional mouthrinses on initial bioadhesion to enamel and dentin in situ. Caries Res..

[28] Meyer F., Enax J. (2019). Hydroxyapatite in oral biofilm management. Eur. J. Dent..

[29] Vukosavljevic D., Hutter J., Helmerhorst E., Xiao Y., Custodio W., Zaidan F., Oppenheim F., Siqueira W. (2014). Nanoscale adhesion forces between enamel pellicle proteins and hydroxyapatite. J. Dent. Res..

[30] Pepla E., Besharat L.K., Palaia G., Tenore G., Migliau G. (2014). Nano-hydroxyapatite and its applications in preventive, restorative and regenerative dentistry: A review of literature. Ann. Stomatol..

[31] Venegas S.C., Palacios J.M., Apella M.C., Morando P.J., Blesa M.A. (2006). Calcium modulates interactions between bacteria and hydroxyapatite. J. Dent. Res..

[32] Al-Ahmad A., Follo M., Selzer A.-C., Hellwig E., Hannig M., Hannig C. (2009). Bacterial colonization of enamel in situ investigated using fluorescence in situ hybridization. J. Med. Microbiol..

[33] Hertel S., Graffy L., Pötschke S., Basche S., Al-Ahmad A., Hoth-Hannig W., Hannig M., Hannig C. (2016). Effect of Inula viscosa on the pellicle’s protective properties and initial bioadhesion in-situ. Arch. Oral Biol..

[34] Hertel S., Pötschke S., Basche S., Delius J., Hoth-Hannig W., Hannig M., Hannig C. (2017). Effect of Tannic Acid on the Protective Properties of the in situ Formed Pellicle. Caries Res..

[35] Jung D.J., Al-Ahmad A., Follo M., Spitzmüller B., Hoth-Hannig W., Hannig M., Hannig C. (2010). Visualization of initial bacterial colonization on dentine and enamel in situ. J. Microbiol. Methods.

